# Androstadienone modulates human aggression in a sex-dependent manner

**DOI:** 10.1093/scan/nsad006

**Published:** 2023-02-15

**Authors:** Yin Wu, Ran Wei, Yu Nan, Yang Hu, Yuting Ye

**Affiliations:** Department of Applied Social Sciences, Hong Kong Polytechnic University, Hung Hom, Hong Kong, China; Research Institute for Sports Science and Technology, Hong Kong Polytechnic University, Hung Hom, Hong Kong, China; Department of Applied Social Sciences, Hong Kong Polytechnic University, Hung Hom, Hong Kong, China; School of Psychology and Cognitive Science, East China Normal University, Shanghai 200062, China; School of Psychology and Cognitive Science, East China Normal University, Shanghai 200062, China; Shanghai Key Laboratory of Mental Health and Psychological Crisis Intervention, East China Normal University, Shanghai 200062, China; Institute of Psychology, School of Public Affairs, Xiamen University, Xiamen 361005, China

**Keywords:** chemosignaling, androstadienone, aggression, dominance, sexual dimorphism

## Abstract

Chemosensory communication is ubiquitous in human social interaction. Androstadienone is a potential candidate human sex pheromone that is associated with social dominance and competition. The aim of the present study was to investigate the effects of androstadienone on aggression. We specifically distinguished two types of aggression, namely proactive and reactive aggression. Two hundred and six male and female participants received either androstadienone or a control carrier in a double-blind, placebo-controlled, between-participants design. Participants performed two aggression tasks, one on reactive aggression and the other on proactive aggression, while they were exposed to the olfactory stimuli. The results revealed that for men, smelling androstadienone reduced both reactive and proactive aggression, whereas it increased reactive aggression in women. These effects were present despite the olfactory stimuli not being explicitly discriminable. These findings provide direct evidence that androstadienone modulates human aggression in a sex-dependent manner.

## Introduction

Aggression is omnipresent in both the animal kingdom and human society. Aggression is defined as any behavior directed toward another individual that is carried out with the intent to cause harm (Anderson and Bushman, 2002). There is increasing recognition that mammalians communicate aggression-relevant information through chemosensory signals, such as body odor and urine (Wyatt, 2003). For example, compared with the effects of urine from socially stable, handled pigs, urine and blood plasma from aggressive pigs reduced aggression in test pigs, highlighting the aggression-influencing properties of urine and other fluids in animals (McGlone *et al.*, 1987). Similarly, odors from the urine of aggressive rather than submissive or castrated male mice can promote aggression in male mice, whereas odors from the urine of normal female mice can inhibit aggression in male mice (Mugford and Nowell, 1970). Furthermore, several chemosignals from mice have been identified as modulating aggressive behavior. Specifically, the protein component of the major urinary protein (MUP) complex, as well as exocrine gland–secreting peptide 1 (ESP1) released into male tear fluids, promotes male–male aggression in mice (Chamero *et al.*, 2007; Hattori *et al.*, 2016).

Like animals, humans can also communicate aggression-related information, such as dominance and competition, through chemosignals. For instance, people are able to judge the personality traits of others based on their body odor such that there is a positive association between self-assessed dominance of odor donors and judgments based on their body odor by others (Sorokowska *et al.*, 2012). Women in the fertile phase of their menstrual cycle prefer body odors of men who score high on trait dominance (Havlicek *et al.*, 2005). Chemosensory signals of competition increase the skin conductance response among perceiving individuals, suggesting that chemosensory signals of competition can be communicated between humans (Adolph *et al.*, 2010). Under the exposure of chemosignals of aggression, receivers adapt an anxiety-related focus in cognition and emotion (Mutic *et al.*, 2016). Chemosignals of a flight response released by a potentially dangerous sender induce an attentional bias (via impaired attentional disengagement) and recruit limbic brain areas such as the thalamus and anterior cingulate cortex in the emotional Stroop task (Mutic *et al.*, 2017). A recent study provided direct evidence on the social chemosignaling of human aggression by showing that hexadecanal, a human body volatile, blocks aggression in men but increases aggression in women (Mishor *et al.*, 2021).

Although the search for human pheromone has not identified undisputed molecular components, previous research suggests that androstadienone (androsta-4,16,-dien-3-one) can be considered as one of the putative human sex pheromones. Androstadienone is present in male semen, axillary hair and the axillary skin surface (Gower and Ruparelia, 1993) and has been shown to increase sympathetic arousal (Bensafi *et al.*, 2003) and cortisol levels (Wyart *et al.*, 2007) and affect the mood (Jacob and McClintock, 2000), probably in a context-dependent manner (Lundstrom and Olsson, 2005). Androstadienone has been shown to convey common information regarding the quality of potential mates such that women’s preference for masculine face shapes is positively correlated with their ratings of androstadienone (Cornwell *et al.*, 2004). Androstadienone can further influence women’s attraction to men. At a speed-dating event, men were judged as more attractive by women who were exposed to androstadienone (Saxton *et al.*, 2008). However, these effects of androstadienone have not been without controversy, as several studies have found that androstadienone does not overtly alter behavior (Lundstrom and Olsson, 2005; Chung *et al.*, 2016; Hornung *et al.*, 2018). Besides the aforementioned empirical research on the role of androstadienone in communicating mating-related information, androstadienone acts as a threatening signal of dominance such that it induces submissive and withdrawal responses during competitive social interaction (Banner and Shamay-Tsoory, 2018) and potentially inhibits competition in men. Moreover, androstadienone increased cooperative behavior between men during decision-making (Huoviala and Rantala, 2013). Note that there are contradictory findings showing that androstadienone increased individualistic responses, while it decreased cooperative responses in men (Banner *et al.*, 2018). Taken together, these studies suggest that androstadienone plays a significant role in human social cognition and decision-making.

In the present study, we investigated the role of androstadienone in human aggression. Aggression is a heterogeneous concept, and here we distinguish two types of aggression, i.e. reactive aggression and proactive aggression (Wrangham, 2018). Reactive aggression is characterized by reacting aggressively to perceived provocation or threats, with the goal being only to remove the provoking stimulus, whereas proactive aggression is a goal-directed and premeditated attack causing harm to another person, with either external or internal reward as a goal (Dodge and Coie, 1987). Notably, these two types of aggression are underpinned by differential neurocognitive mechanisms (Nelson and Trainor, 2007). Specifically, reactive aggression engages neural circuits that are associated with emotional reactivity, emotion regulation and cognitive control, such as the amygdala and orbitofrontal cortex (Fanning *et al.*, 2017), while proactive aggression is strongly associated with the left dorsolateral prefrontal cortex and medial prefrontal cortex, brain regions implicated in instrumental motivation (Zhu *et al.,* 2022). Moreover, reactive aggression has been related to increased sympathetic reactivity, while proactive aggression has been related to hypoarousal of the sympathetic and parasympathetic nervous systems in men and augmented parasympathetic nervous system activity in women, suggesting the dissociable psychobiological profile of these two types of aggression (Thomson *et al.*, 2021). Thus, it is necessary to examine the effects of androstadienone on them separately, in view of the reported effects of androstadienone on emotion perception and sympathetic arousal (Bensafi *et al.*, 2003; Ye *et al.*, 2019). Since previous research has identified a sexual dimorphic feature of androstadienone (Savic *et al.*, 2001; Zhou *et al.*, 2014; Ye *et al.*, 2019) and aggression (Hay, 2007), here we examined the effects on both men and women. We hypothesized that the effects of androstadienone on human aggression would be sex-specific.

## Materials and methods

### Participants

A total of 206 healthy non-smokers (106 men, mean age = 22.15 years, s.d. = 2.43, range = 18–34; and 100 women, mean age = 20.50 years, s.d. = 1.69, range = 18–29 years) were recruited for this study. All participants reported to be Han Chinese and heterosexual (Kinsey score = 0) and have normal or corrected-to-normal vision, a normal sense of smell and no respiratory allergy or upper respiratory infection. The sample size was determined based on the effect size reported in previous studies (Mutic *et al.*, 2016), which tested the effects of aggression-related chemosignals. Using G*Power 3.1, we set α to 0.05 and power to 0.95, resulting in a sample size of 42 in each subgroup (per sex per olfactory condition). We recruited 206 participants to allow for possible non-compliance or impossibility of model fit. All female participants were tested around the periovulatory phase of their menstrual cycles (i.e. at the midpoint of their menstrual cycle, ∼14 days from the onset of their last period of a normalized 28-day cycle)^1^, which was determined by self-reporting. Participants were randomly assigned to either androstadienone or the control solution in a double-blind, between-participants design (men: 56 in the androstadienone and 50 in the control condition and women: 49 in the androstadienone and 51 in the control condition). All procedures were approved by the local research ethics committee and were in accordance with the Declaration of Helsinki. All participants provided written informed consent and were paid RMB 50 (∼$7.86) as a flat fee.

### Olfactory stimuli

The olfactory stimuli consisted of androstadienone (500 μM in 1% v/v clove oil propylene glycol solution) and the carrier solution alone (control, 1% v/v clove oil in propylene glycol). The concentration of androstadienone used in the current study (500 μM) was comparable to that in freshly produced apocrine sweat (mean = 0.44 nmol/μl = 0.44 × 10^−3^ mol/l = 440 μM), hence arguably ecologically relevant (Gower *et al.*, 1994). The stimuli were presented in identical 40 ml polypropylene jars, each containing 5 ml of clear liquid, and connected with two Teflon nosepieces via a Y-structure. The distinguishability of the olfactory stimuli was assessed in an independent sample of 85 healthy non-smokers (41 men and 44 women, mean age = 21.18 years, s.d. = 1.94). Participants were asked to complete six trials of a triangle odor discrimination task. In each trial, they were presented with three smells, two identical (control) stimuli and another different (target) stimulus. Participants were asked to report which one was the odd one out. Each of the three olfactory stimuli served as the target once and as the control once. Thus, the probability of arriving at a correct response by chance was one-third. Here, participants were at the chance level in discriminating the olfactory stimuli [mean accuracy ± s.d. = 0.35 ± 0.24 *vs* chance (0.33), *P* = 0.51], and there was no reliable difference in perceived intensity [*t(84)* = 1.47, *P *= 0.14] or pleasantness [*t(84)* = 0.29, *P *= 0.77].

### General procedure

Upon arrival, participants completed a battery of questionnaires, including the Buss–Perry Aggression Questionnaire (Buss and Perry, 1992), the Reactive–Proactive Aggression Questionnaire (Raine *et al.*, 2006), the Barratt Impulsiveness Scale (BIS-11) (Patton *et al.*, 1995) and the Liebowitz Social Anxiety Scale (Liebowitz, 1987), which measured trait aggression, proactive and reactive aggression, impulsiveness and trait social anxiety, respectively. Before and after smelling the olfactory stimuli, participants also completed the Positive and Negative Affect Schedule to measure changes in emotional states (Watson *et al.*, 1988).

We employed a between-participants design in which participants were randomly assigned to either androstadienone or the control solution. Participants were asked to complete the Taylor Aggression Paradigm (TAP) (Taylor, 1967) first and then the Reward Interference Task (RIT) (Zhu *et al.*, 2019), during which they were continuously exposed to either androstadienone or the control solution alone. There was a break of 15–20 min between the two tasks, in which no olfactory stimuli were presented. Participants were asked to hold the jar with their non-dominant hand, position the nosepieces inside their nostrils and continuously inhale through the nose and exhale through the mouth while performing the experimental tasks. The experimenter was blind to the identity of the olfactory stimuli and was not in the testing room while the participants performed the tasks. To ensure the participants followed the experimental instructions, their activities were continuously monitored via a video monitor by the experimenter sitting in the adjacent room.

### Experimental tasks

#### Taylor Aggression Paradigm

Reactive aggression was measured by using the TAP task (Taylor, 1967), in which the participants were convinced that they were competing against an opponent of the same sex and the winner could exert a high or low punishment against the loser of the current round of the competition. At the start of the task, the participant received RMB 10 as an endowment. A high punishment refers to the loudest bearable aversive noise (75 dB) and a loss of RMB 0.5, while a low punishment refers to the lowest audible noise (15 dB) and a loss of RMB 0.1. The duration of the noise punishment was 3.2 s.

**Fig. 1.**
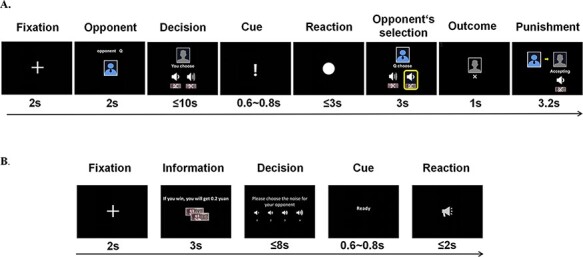
(A) Trial timing for the TAP. (B) Trial timing for the RIT.

The TAP task comprised two blocks. Each block contained 20 rounds, with half against a low-provoking opponent (labeled as ‘Q’) and the other half against a high-provoking opponent (labeled as ‘W’). The sequence of rounds in each block was pre-determined by a computer program such that no opponent would appear more than three times in a row. Each trial started with informing the participant that one opponent had been randomly selected (see Figure 1A). The participant and his/her opponent were asked to choose the intensity of the punishment within 10 s. On the reaction-time task screen, the participant and his/her opponent were asked to press the space bar as soon as possible, and the player who responded faster was the winner. Participants were shown the intensity of the punishment selected by the opponent before showing the outcome of the present trial. In the end, the corresponding punishment was delivered to the participant if he/she failed. It took ∼5.5 min to finish each block, with a 2-min interval between blocks, during which participants were not exposed to the olfactory stimuli.

The punishment selected by the opponent and the outcome of the competition were pre-determined by an algorithm such that the probability of loss against either the low- or high-provoking opponents was 50% and the percentages of high punishment chosen by the high- and low-provoking opponents were 80% and 20%, respectively.

#### Reward Interference Task

Proactive aggression was measured by using a modified RIT (Zhu *et al.*, 2019), in which participants could select a certain level of noise to interfere with his/her opponent’s performance and win the competition, while their opponent had no such option (see Figure 1B). The opponent was fictitious, but the participants were convinced that they were competing with someone of the same sex. Therefore, there were two players in the task, player A (the participant) and player B (whose responses were pre-determined by a computer program). Player A could select a certain level of noise (four levels: 1 = 80 dB, 2 = 90 dB, 3 = 100 dB and 4 = 110 dB) with which to interfere with his/her opponent’s performance in the subsequent reaction-time task. In the reaction-time task, the person who responded faster was the winner and won the amount of money as indicated at the beginning of the trial. Thus, the noise selected by player A would interfere with the opponent’s performance and was used as an index of proactive aggression. Each trial started by presenting the participant with the amount of monetary reward for the winner, and then the participant could choose the noise for the opponent. In the reaction-time task, participants were asked to respond as quickly as possible, and they were told that the noise selected would be delivered to the opponent.

There were 44 trials in the RIT, with 20 trials for the high-reward condition (RMB 1–5), 20 trials for the low-reward condition (RMB 0.1–0.5) and 4 trials for the filler condition (no reward). The probability of winning/losing in each trial was pre-determined at the chance level, i.e. 50%, and the outcome of each trial was not shown immediately. Only two trials were randomly selected, and the final payment for this task was randomly determined in the range between 10 and 15 RMB. It took ∼11.5 min to finish the task.

### Statistical analyses

All the data were tested for normality before further analyses. For the TAP task, the percentage of choice of high punishment in each condition was used as the dependent variable. The dependent variable was analyzed using analysis of variance (ANOVA) with the opponent (high *vs* low provoking) as the within-participants factor and sex (man *vs* woman) and olfactory condition (androstadienone *vs* control) as two between-participants factors. For the RIT, the dependent variable was the mean of selected level (*M*) and was analyzed using ANOVA with reward magnitude (high *vs* low) as the within-participants factor and sex (man *vs* woman) and olfactory condition (androstadienone *vs* control) as two between-participants factors. For each task, interactions between two between-participants factors were decomposed by performing ANOVAs with opponent (TAP task)/reward (RIT) as the within-participants factor and olfactory condition as the between-participants factor in each sex separately. To exclude the possibility that any olfactory effect on aggression was due to individual differences in personality traits, we ran a series of analyses to check the robustness of the findings in the main analysis. First, we compared group differences on the trait measures. Second, we included these scores as covariates in the statistical model to test if the effects of interest (interactions between olfactory condition and sex) remained significant.

## Results

The distributions of data from both tasks were verified to be normal by using the Shapiro–Wilk test (*Ps* > 0.05). We first looked at sex differences in the baseline aggression level among the control group participants. While men (*M *= 0.53, s.d. = 0.23) showed increased reactive aggression compared with women [*M *= 0.39, s.d. = 0.23; *t(99) *= 3.01, *P *= 0.003, Cohen’s *d* = 0.60], men (*M *= 2.64, s.d. = 0.68) and women (*M *= 2.47, s.d. = 0.65) did not differ in their baseline proactive aggression level [*t(99) *= 1.32, *P *= 0.19].

### Androstadienone reduced reactive aggression in men, while it increased reactive aggression in women

**Fig. 2.**
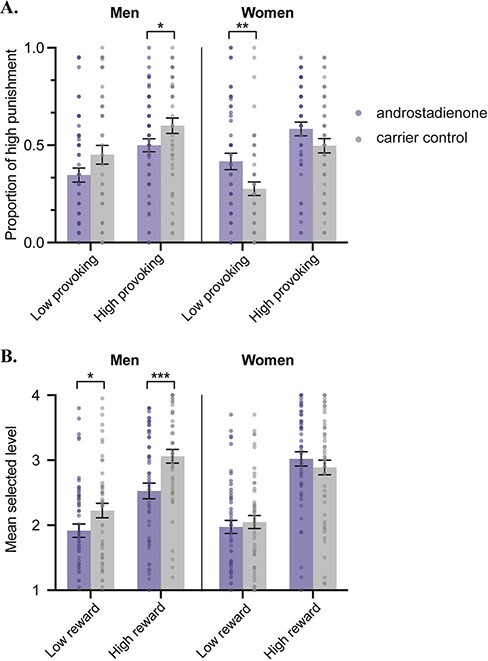
(A) Androstadienone reduced reactive aggression in men, while it increased reactive aggression in women. (B) Androstadienone only reduced proactive aggression in men. *P ≤ 0.05, **P ≤0.01 and ***P ≤ 0.001.

An omnibus ANOVA with opponent type and olfactory condition as the within-participants factors and sex of the group as the between-participants factor revealed a significant main effect of opponent type [*F* (1, 202) = 61.96, *P *< 0.001, partial η^2^ = 0.24], without interaction with olfactory condition or the sex of the group (*P*s > 0.33). Specifically, participants were more likely to choose high punishment when facing a high-provoking opponent (*M* = 0.54, s.d. = 0.26) rather than a low-provoking opponent (*M* = 0.37, s.d. = 0.30). On top of that, there was a significant two-way interaction between olfactory condition and sex [*F* (1, 202) = 11.73, *P *< 0.001, partial η^2^ = 0.055], suggesting androstadienone affected the reactive aggressive behaviors in men and women differently. Further analysis revealed that exposure to androstadienone (*M* = 0.42, s.d. = 0.20) reduced reactive aggression compared with exposure to the control solution among men [*M* = 0.53, s.d. = 0.23, *F* (1, 104) = 5.93, *P *= 0.017, partial η^2^ = 0.054], while it increased reactive aggression in women (androstadienone 0.50 ± 0.24 *vs* control 0.39 ± 0.23) [*F* (1, 98) = 5.79, *P *= 0.018, partial η^2^ = 0.056] (see Figure 2A).

In each sex group, the two-way interaction between the opponent and olfactory condition was not significant, both *P*s > 0.1, indicating that the effects of odor were comparable when facing high- and low-provoking opponents.

### Androstadienone reduced proactive aggression only in men

We conducted an omnibus ANOVA with reward type and olfactory condition as the within-participants factors and sex of the group as the between-participants factor. The results identified a significant main effect of reward magnitude [*F* (1, 202) = 264.42, *P *< 0.001, partial η^2^ = 0.57], as well as a significant interaction between reward and sex [*F* (1, 202) = 4.59, *P *= 0.033, partial η^2^ = 0.022]. Specifically, although high reward increased proactive reward in both sexes (*P*s < 0.001), the difference of selected level between high and low reward was larger in women (*M* = 0.94, s.d. = 0.73) than in men (*M* = 0.72, s.d. = 0.74).

As for the effect of androstadienone, we found a significant two-way interaction between olfactory condition and sex [*F* (1, 202) = 5.49, *P *= 0.020, partial η^2^ = 0.026], suggesting dissociable effects of androstadienone on men and women.

Moreover, there was a significant three-way interaction between reward magnitude, olfactory condition and sex [*F* (1, 202) = 4.47, *P *= 0.036, partial η^2^ = 0.022]. The interaction was decomposed by looking at the effects of reward magnitude and olfactory condition in each sex separately (see Figure 2B). Among men, androstadienone (*M* = 2.22, s.d. = 0.75) reduced proactive aggression compared with the control solution [*M* = 2.64, s.d. = 0.68; *F* (1, 104) = 9.16, *P = *0.003, partial η^2^ = 0.081]. Nevertheless, the interaction between reward magnitude and olfactory condition was not significant [*F* (1, 104) = 2.48, *P = *0.12], suggesting that androstadienone exerted comparable effects in both high- and low-reward conditions. For women, neither the main effect of olfactory condition nor the interaction between olfactory condition and reward magnitude was significant (both *P*s > 0.15).

### Personality characteristics and emotional states did not account for the androstadienone effect

To investigate whether the androstadienone-induced changes in aggressive behavior could be accounted for by other potential contributing factors, we compared the differences in trait aggression, trait reactive and proactive aggression, trait impulsiveness and trait social anxiety between the two olfactory groups in each sex. We found no significant differences in these measures between the two groups (see Table S1).

Next, to rule out the possibility that individual differences in personality traits and changes in emotional states could confound the behavioral effects observed, we included these variables as covariates in the statistical model. The pattern of results of the variables of interest was essentially the same with our original model, as the interaction between olfactory condition and sex remained significant (for reactive aggression, β* = *−0.21*, Z = *−3.33*, P = *0.001; and for proactive aggression, β* = *−0.48*, Z = *2.50*, P = *0.01) (see Tables S2 and S3). Thus, the effects of androstadienone on aggression could not be attributed to group differences in personality traits and changes of emotional states.

## Discussion

In the present study, we investigated the effects of androstadienone on reactive and proactive aggression in both men and women. At the baseline level, men exhibited stronger reactive aggression compared with women, corroborating past findings that men are more responsive to provocation (Eagly and Steffen, 1986). However, men and women did not differ in proactive reaction in our data. Most importantly, we found that sniffing androstadienone inhibited both reactive and proactive aggression in men, while it promoted reactive aggression in women, adding to the growing literature finding that chemosensory cues can modulate interpersonal interaction. These effects were present despite the olfactory stimuli being not explicitly discriminable. Moreover, the effects were independent of participants’ personality traits and changes of emotional states. Note that in the present study, participants were asked to perform the reactive aggression task first and then the proactive aggression task, with a break of 15–20 min between the two tasks. It is unclear if there was any interaction between task order and treatment or between task order, treatment and sex. Future research needs to counterbalance the order of the two tasks between participants to exclude possible carryover effects.

As a fundamental social behavior, aggression is vital for survival and reproduction in both sexes. However, research across species shows that it is often displayed differentially between both sexes as a result of natural selection, usually at a higher level in men than women, accompanied by differential neural circuits (Hashikawa *et al.*, 2018; Pandolfi *et al.*, 2021). In line with evidence from animals, aggression studies in humans indicate that while men tend to express physical, overt and direct aggression, women tend to express relational and indirect aggression more often (Archer and Coyne, 2005; Gregoski *et al.*, 2005). Specifically, men are more likely to exhibit a higher level of reactive aggression than women (Bettencourt and Miller, 1996). Although studied to a lesser extent, proactive aggression has been reported to be comparable between men and women (Im *et al.*, 2018; Boccadoro *et al.*, 2021). In line with these findings, our results showed that men have higher reactive aggression compared with women, while men exhibited comparable levels of proactive aggression with women. Note that there is also contrasting evidence suggesting significant sex differences in proactive aggression rather than reactive aggression (Maneiro *et al.*, 2022).

On top of the sexual dimorphism in aggression, the sex-specific effect of androstadienone adds to the growing literature the sexual dimorphic nature of this chemosignal. For example, in a gender identification task where participants were asked to judge the gender of visually presented point-light walkers, smelling androstadienone biased heterosexual women, but not men, toward perceiving the walkers as more masculine (Zhou *et al.*, 2014). Furthermore, androstadienone biased heterosexual women, but not men, toward perceiving the male walkers as happier and more relaxed and female walker as sadder, possibly facilitating sex-appropriate behavior and promoting intrasexual competition, respectively. (Ye *et al.*, 2019). Brain imaging studies have revealed sex-dissociated neural responses such that androstadienone activates the hypothalamus in women rather than in heterosexual men (Savic *et al.*, 2001). As with evidence from both animal and human studies, this study suggests that androstadienone modulates human aggression in a sex-dependent manner, which has been demonstrated in social odor communication among most mammals (Chamero *et al.*, 2007; Mishor *et al.*, 2021). The ecological relevance of these results is open to multiple interpretations, but they may suggest androstadienone as a putative sex pheromone signaling masculinity and dominance. In particular, androstadienone promotes intrasexual competition for potential mates in women, while it inhibits aggressive behavior toward dominant individuals in men.

In men, androstadienone acts as a chemosignal of dominance that induces behavioral avoidance and social withdrawal behaviors. For instance, androstadienone reduces interference in the processing of angry target faces by non-relevant emotional words, suggesting androstadienone could prepare individuals for an upcoming conflict by highlighting the threatening facial expressions (Hornung *et al.*, 2017). Androstadienone enhances men’s judgment of others regarding one’s hierarchical ranking and increases gaze avoidance of dominant poses (Banner and Shamay-Tsoory, 2018), highlighting the role of androstadienone in dominance perception. In a competitive decision-making task, smelling androstadienone induces more avoidance tendencies during social interaction (Banner *et al.*, 2018). The chemosignal communication of dominance by androstadienone could underlie our findings that both reactive and proactive aggression were significantly reduced under the exposure of androstadienone in men.

Note that Banner *et al.* (2018) did not observe a significant effect of androstadienone on aggressive responses in their studies. According to a previous work (Dodge and Coie, 1987), proactive aggression tends to be driven by low emotional arousal and high levels of instrumentality to obtain reward or benefits in the absence of provocation, whereas reactive aggression is characterized by negative affect and emotional response, which makes individuals respond impulsively after provocation. In the settings of Banner *et al.* (2018), aggressive response was defined by decisions leading to monetary loss to others coupled with no gain or loss to the participants themselves. In their study, there was neither direct provocation nor explicit reward associated with behavior, and thus the decisions could not be termed as either reactive or proactive aggression based on the definition presented by Dodge and Coie (Adolph *et al.*, 2010). Nevertheless, we encourage future research to investigate whether the effects of androstadienone on withdrawn or submissive motivations in men further depend on social or situational factors.

Preferential processing of male aggression signals has been shown in women at the neural level. Specifically, women demonstrate enhanced neural processing of human aggression sweat from men rather than that from women. The increased sensitivity to male aggression signals has adaptive value since male aggression typically targets physical harm, in particular toward women (Pause *et al.*, 2020). Furthermore, women have been reported to orient their attention toward other women and their emotional perception of other women is more negative under exposure to androstadienone (Parma *et al.*, 2012; Ye *et al.*, 2019; Pause *et al.*, 2020), which may mediate intrasexual competition. In accordance with these findings, our findings provide the first evidence that women respond to androstadienone by increasing their reactive aggression when confronting provocation, which is likely to facilitate intrasexual competition.

Interestingly, for women, only reactive aggression was affected under exposure to androstadienone. First, a previous study has shown that for women, reactive aggression is related to a heightened sympathetic nervous system, while proactive aggression is associated with an augmented parasympathetic nervous system (Thomson *et al.*, 2021). Moreover, androstadienone has been found to heighten sympathetic arousal in women but decrease it in men (Bensafi *et al.*, 2003; Wyart *et al.*, 2007), which may underlie the divergent effects of androstadienone on reactive aggression we found here. The increased reactive aggression could adaptively prepare women for threat so as to remove the provoking stimulus (Wrangham, 2018). Second, previous research has shown that female aggression is mostly indirect, and women are less physically and verbally aggressive than men when unprovoked, possibly due to social and cultural norms (Bettencourt and Miller, 1996). This could possibly account for the lack of an androstadienone effect on proactive aggression in women.

Some issues warrant further discussion. First, previous research has shown that androstadienone has divergent effects on homosexual and heterosexual individuals (Savic *et al.*, 2005; Zhou *et al.*, 2014). Future studies testing homosexual individuals could help to further clarify the potential role of androstadienone as a human sex chemosignal. Second, in this study, we only investigated aggression toward intrasexual individuals (i.e. women to women and men to men). Evidence from rodents has shown that aggressive behaviors depend on the sex of opponents, as male mice aggress mostly toward male mice, but not female mice, and rarely toward castrated male mice (Xu *et al.*, 2012; Trouillet *et al.*, 2019). Furthermore, previous research has shown that the effects of androstadienone are contingent upon not only the recipients’ own sex but also their sex perception of other individuals, which ensures sex-appropriate behavior (Ye *et al.*, 2019). Hence, it would be interesting to investigate the effects of androstadienone on intersexual aggression, e.g. men to women and women to men. Third, the present study was not pre-registered, and we encourage future research to adopt an open-science approach and pre-register the research hypothesis, sample size and analytical plan.

In conclusion, our findings demonstrate that while androstadienone decreases both proactive and reactive aggression in men, it increases reactive aggression in women. These data provide direct evidence that androstadienone modulates human aggression in a sex-dependent manner.

## Supplementary Material

nsad006_Supp

## Data Availability

All data and analysis scripts are available on the project’s Open Science Framework (OSF) page: https://osf.io/rgta8/.

## Footnotes

1 We included women’s self-reported days from the onset of their last period of a normalized 28-day cycle as a covariate in the statistical models; the pattern of results regarding the effects of androstadienone on reactive/proactive aggression remained the same.
