# Supplementary material for: Androstadienone modulates human aggression in a sex-dependent manner
Source: Soc Cogn Affect Neurosci. 2023 Feb 15;18(1):nsad006. doi: 10.1093/scan/nsad006 (PMC9960015; doi:10.1093/scan/nsad006)
Supplement: nsad006_Supp [file nsad006_supp.zip › scan-22-085-File004.docx]

Table S1. Personality differences between androstadienone and control groups.

|  | Androstadienone group  (M ± SD) | Control group  (M ± SD) | *t* | *p* |
| --- | --- | --- | --- | --- |
| **Buss–Perry Aggression Questionnaire** |  |  |  |  |
| Men | 30.00±10.24 | 32.78±9.77 | -1.43 | 0.16 |
| Women | 29.69±14.27 | 30.69±13.02 | -0.36 | 0.72 |
| **Barratt Impulsiveness Scale** |  |  |  |  |
| Men | 62.00±7.56 | 64.00±7.13 | -1.40 | 0.17 |
| Women | 64.29±8.02 | 65.49±8.70 | -0.72 | 0.47 |
| **Reactive-Proactive Aggression Questionnaire - proactive aggression** |  |  |  |  |
| Men | 14.63±4.17 | 15.86±4.49 | -1.47 | 0.15 |
| Women | 14.20±6.14 | 13.24±3.34 | 0.97 | 0.33 |
| **Reactive-Proactive Aggression Questionnaire - reactive aggression** |  |  |  |  |
| Men | 24.21±6.79 | 26.14±5.75 | -1.57 | 0.12 |
| Women | 26.35±7.50 | 27.31±7.75 | -0.63 | 0.53 |
| **Liebowitz Social Anxiety Scale** |  |  |  |  |
| Men | 43.64±19.90 | 48.82±23.70 | -1.22 | 0.22 |
| Women | 47.92±22.04 | 48.78±22.21 | -0.20 | 0.85 |

Note. Reactive-Proactive Aggression Questionnaire (proactive aggression), proactive aggression subscale of the Reactive-Proactive Aggression Questionnaire; Reactive-Proactive Aggression Questionnaire (reactive aggression), reactive aggression subscale of the Reactive-Proactive Aggression Questionnaire.

**Table S2.** Results of regression analysis examining the impacts of olfactory condition and control variables on reactive aggression.

|  | *β* | *t* | *p* |
| --- | --- | --- | --- |
| Sex | 0.06 | 1.34 | 0.18 |
| Olfactory condition | 0.09 | 1.96 | 0.05* |
| Total score on RPQ-reactive aggression | 0.003 | 1.21 | 0.23 |
| Total score on BPAQ | 0.004 | 2.51 | 0.01** |
| Total score on BIS-11 | 0.002 | 0.86 | 0.39 |
| Total score on LSAS | -0.0006 | -0.24 | 0.81 |
| Changes in positive affect | -0.002 | -0.64 | 0.53 |
| Changes in negative affect | -0.002 | -0.62 | 0.54 |
| Sex*Olfactory condition | -0.21 | -3.33 | 0.001*** |

Note. RPQ-reactive aggression, reactive aggression subscale of the Reactive-Proactive Aggression Questionnaire; **p* ≤ 0.05, ***p* ≤0.01, ****p* ≤ 0.001.

**Table S3.** Results of regression analysis examining the impacts of olfactory condition and control variables on proactive aggression.

|  | *β* | *t* | *p* |
| --- | --- | --- | --- |
| Sex | 0.36 | 2.57 | 0.01** |
| Olfactory condition | 0.44 | 3.26 | 0.001*** |
| Total score on RPQ-proactive aggression | -0.0008 | -0.066 | 0.95 |
| Total score on BPAQ | 0.006 | 1.38 | 0.17 |
| Total score on BIS-11 | -0.009 | -1.27 | 0.20 |
| Total score on LSAS | -0.0009 | -0.39 | 0.70 |
| Changes in positive affect | 0.01 | 1.53 | 0.13 |
| Changes in negative affect | -0.006 | -0.69 | 0.49 |
| Sex*Olfactory condition | -0.48 | -2.50 | 0.01** |

Note. RPQ-proactive aggression, proactive aggression subscale of the Reactive-Proactive Aggression Questionnaire; ***p* ≤0.01, ****p* ≤ 0.001.
